# How to make science more efficient

**DOI:** 10.7554/eLife.106819

**Published:** 2025-03-27

**Authors:** Stuart Buck

**Affiliations:** 1 Good Science Project Atlanta United States

**Keywords:** Science Under Threat in the United States, science, politics, science funding, science policy, National Institutes of Health, None

## Abstract

DOGE needs to completely rethink its efforts to increase the efficiency of the federal agencies that fund research in the US.

When the Trump administration tasked the entrepreneur Elon Musk with establishing a new Department of Government Efficiency (DOGE), I was cautiously optimistic.

As executive director of the Good Science Project, I have spent several years writing about the lamentable fact that, according to multiple government-sponsored surveys, researchers are spending more and more of their time on admin, and less on research. As the Federal Demonstration Partnership (FDP) reported in 2020: “the trend seems to be that **time taken from research by requirements is increasing, not decreasing**. PIs reported that almost half of their available research time for federal projects had to be allocated to fulfilling requirements instead of focusing on the content of their research projects.”

Virtually no one thinks that we are making the best use of scientists’ time. We would be better off if institutions such as the National Institutes of Health (NIH) and the National Science Foundation (NSF) could operate more nimbly, with less bureaucracy, and with more flexibility for scientists to engage in truly innovative work. A national effort to streamline government funding would be welcome to most scientists.

At least, such was my initial hope for DOGE. In reality, however, the initial months of DOGE were almost entirely contradictory to its putative aims. Hundreds of federal research grants from the NIH were canceled for being about transgender issues, or about marijuana, or about diversity. Moreover, over a thousand people who worked at NIH (including at least 20 who worked on preventing lab leaks!) and hundreds at NSF were fired for no reason other than that it was possible to do so under federal civil service regulations because they were “probationary” (either as a new hire or as someone who had recently been promoted). Some of the NSF employees were then rehired following a court order. And an attempt by DOGE to limit the indirect costs paid on NIH grants to 15% was immediately blocked by a federal court because it was inconsistent with federal rules and Congressional mandates.

Whatever one thinks about diversity efforts, the overall level of federal employment or indirect costs, *none* of these DOGE actions have anything whatsoever to do with making life more efficient for American scientists. That much is plain.

If we wanted to improve government efficiency, we should start by looking at a chart complied by the COGR – an organization that represents research universities, affiliated medical centers, and independent research institutions – that tracks the number of regulations and policies that apply to federally-funded researchers: this number has increased from 16 in 2000 to 270 today. I am old enough to remember the year 2000, and I don’t recall that federally-sponsored research was in such dire straits that the number of regulations governing it needed to be multiplied by a factor of sixteen.

In short, if DOGE wants to fulfill the original mission to reduce bureaucracy and improve efficiency, it needs to execute a 180 degree change of direction. It needs to start consulting with experts who have been tracking the administrative burden on science for decades (such as those at COGR and the FDP). It should then work collaboratively with the White House Office of Management and Budget, science agencies, and universities in a commonsense effort to streamline proposals, progress reports, budgets, and other regulations (including those related to Institutional Review Boards and Institutional Animal Care and Use Committees). More than anything that DOGE has done to date, such an effort would ensure that researchers would be able to spend much more of their time doing what they are supposed to be doing – research.

